# Exploring jump back behavior patterns and reasons in e-book system

**DOI:** 10.1186/s40561-021-00183-6

**Published:** 2022-01-04

**Authors:** Boxuan Ma, Min Lu, Yuta Taniguchi, Shin’ichi Konomi

**Affiliations:** 1 Graduate School of Information Science and Electrical Engineering, Kyushu University, Fukuoka, Japan; 2 Faculty of Arts and Science, Kyushu University, Fukuoka, Japan; 3 Research Institute for Information Technology, Kyushu University, Fukuoka, Japan

**Keywords:** Reading behavior, E-book event stream, Educational big data, Jump-back

## Abstract

With the increasing use of digital learning materials in higher education, the accumulated operational log data provide a unique opportunity to analyzing student learning behaviors and their effects on student learning performance to understand how students learn with e-books. Among the students’ reading behaviors interacting with e-book systems, we find that jump-back is a frequent and informative behavior type. In this paper, we aim to understand the student’s intention for a jump-back using user learning log data on the e-book materials of a course in our university. We at first formally define the “jump-back” behaviors that can be detected from the click event stream of slide reading and then systematically study the behaviors from different perspectives on the e-book event stream data. Finally, by sampling 22 learning materials, we identify six reading activity patterns that can explain jump backs. Our analysis provides an approach to enriching the understanding of e-book learning behaviors and informs design implications for e-book systems.

## Introduction

Learning Management Systems (LMSs) and e-book systems are increasingly used together for supporting daily classroom teaching. Recently, many countries plan to use e-books in schools, and many traditional textbooks have been replaced by e-books in many schools (Rainie et al., 2012). For example, The Korean Education and Research Information Service announced a digital textbook usage plan in 2007 (Shin, 2012), while the Japanese government is scheduled to change all textbooks for elementary, middle, and high schools into digital textbooks by 2020 (Yin et al., 2014).

Compared with traditional textbooks, e-books have benefited from a lower price, greater interactivity, being quickly obtained from the Internet, taking up less space, and being more portable (Shepperd et al., 2008). Most importantly, digital textbook systems allow it to collect operational log data regarding the students’ reading processes which is not possible with traditional textbooks. By using e-books, a significant amount of logged educational data can be created. These log data are a recording of learning practices (Mostow, 2004) and represent one of the most valuable sources of information for analyzing the activities of students. Although such an approach cannot capture the totality of reading experiences, it allows for the detailed and precise recording of e-book navigation and interaction. Reading learning materials is one of the most common and essential activities in current college education. Actually, the majority of the time that students spend in class is related to the reading of provided learning materials such as teaching slides. Analyzing the data from e-book systems provides a novel and great potential for understanding students’ behaviors and enhancing education delivery. Recently, many works have been conducted on the click-level interactions between users and the e-book systems to understand better how students learn and what they need when reading learning materials (Crossley et al., 2016; Ma et al., 2020; Oi et al., 2015; Shimada et al., 2016).

**Fig. 1:**
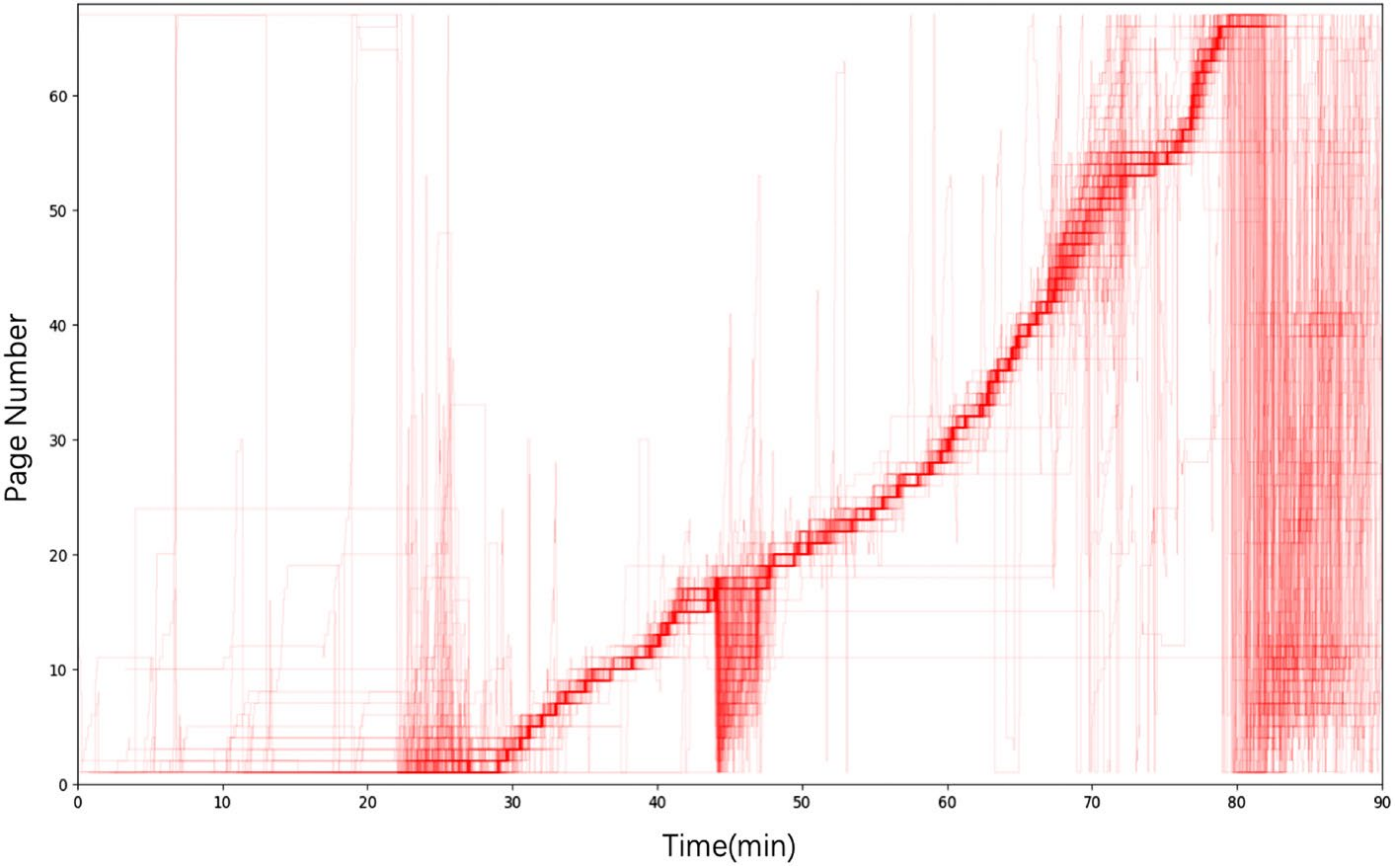
Students’ page flip patterns across the lecture

Among many reading behaviors, jump back is a frequent behavior with strong user intention in E-book learning (McKay, 2011). Previous works find that learners go back to the previous pages many times for reviewing or reflecting (Yin et al., 2015b). Figure 1 presents the visualization of students’ page flip patterns in one of the lectures of our dataset. The intersection of the time and page shows the current page that the student was viewing at a specific time and each line shows the reading sequence of a particular student. It shows jump back behaviors occurs many times during the class. According to the literature, the reasons for jump-backs may include that the students want to view the difficult or missed content that is found not understood well when viewing later pages, or the students simply refer to the related content when doing quizzes or practices, etc. (Ren et al., 2017; Yin et al., 2019). This action can be linked to a review learning strategy, which allots time to commit information to long-term memory (Lindsey et al., 2014). It can also be linked to a reflection learning strategy, which involves linking current knowledge to previous knowledge (Costa & Kallick, 2008). Freeman and Saunders (2016) modeled five main jump categories in E-book reading, including forward with no jumps (FOR), small jump forward (SJF), big jump forward (BJF), small jump back (SJB), and big jump back (BJB). In addition, Chen et al. (2021) found jump-back behavior is significantly positively correlated to academic achievement.

However, many jump behaviors in the e-book are due to the lack of overview and inadequate navigation features. It is difficult to jump to the right page once when reading a book on a digital device and learners usually jump several times to find the correct position (Myrberg, 2017). Although several previous studies have investigated the jump back behaviors, their methods did not consider such multiple jump behaviors that are not explicitly reflected from the log data. In addition, they did not connect jump behaviors and learning content. Therefore, this study conducts a systematic study as a first step to investigate jump-backs and identify the reasons that can lead to a jump back in an actual classroom setting by using students’ reading logs that were collected from a digital textbook reader. Our research questions are as follows:

- RQ1. General Characteristics: What are the general characteristics of students’ jump-back behaviors? How do the general characteristics vary in different slides? How do the general characteristics vary in and out of class?
- RQ2. Student preferences: Do students have personal preferences when they jump back? Can we find students’ possible jump-back patterns in and out of class?
- RQ3. Relations to student learning achievements: Are there any relationships between students’ jump-back behaviors and learning achievements?
- RQ4. Causes for jump-back behaviors: Why jump back behaviors occur? Can we identify the reasons that can lead to a jump back?

This paper studied the student jump-back behaviors and intentions. Through the analytics of e-book event stream data, we first formally define the different types of “jump-back” related behaviors. Then, we studied the jump-back behaviors by employing statistical analysis methods as well as the k-means clustering algorithm to answer the questions above from different perspectives. Finally, our analysis provides a rich understanding of e-book learning and informs design implications for future e-book systems and tools.

## Related work

Analyzing learning behavior patterns is critical for fostering a better understanding of learning processes and optimizing learning and its environments (Liu et al., 2017). Learning behavior can be considered as a series of actions that learners produce during the learning process, including reading textbooks, answering quizzes, watching videos, browsing forums, uploading resources, accessing learning platforms, discussing and communicating with others, and so on (Akçapinar et al., 2020; Lorenzen et al., 2018). Researchers have indicated that the analysis of user behaviors and experience can facilitate the design of learning systems, materials, or activities (Law & Lárusdóttir, 2015; Sutcliffe & Hart, 2017).

Many researchers focus on studying user behavior patterns and their implications from the log data recorded when learners interact with learning materials like videos. For example, identify student behavior patterns based on features of available interaction types, i.e., pausing, forward, backward seeking, and speed changing (Kim et al., 2014b; Li et al., 2015); analyzing correlations between user behaviors and video content (Avlonitis & Chorianopoulos, 2014; Chorianopoulos, 2013; Chorianopoulos et al., 2011). In general, the literature has pointed to interesting findings for analysis of video learning behaviors and provides insights for the analysis of e-book learning behaviors. However, it is limited to video analytics in MOOCs, which may significantly differ from the e-books in university environments.

Recently, a few pieces of research have been conducted on the click-level interactions between users and the e-book systems to understand better how students learn and what they need when reading learning materials. A previous study that analyzed students’ digital textbook interaction data indicates that the course outcome is directly related to the reading of a textbook (Akçapınar et al., 2019). Others include pattern mining of preview and review activities (Oi et al., 2015), understanding page-flip behavior of students (Ma et al., 2020; Yin et al., 2015a), browsing pattern mining (Shimada et al., 2016), analysis of highlighters on e-textbooks (Taniguchi et al., 2019), predict of the student’s performance (Brinton & Chiang, 2015; Okubo et al., 2017), predict of the class completion (Crossley et al., 2016), and clustering learner behaviors (Wang et al., 2016). Cheng and Tsai (2014) collected video-recorded data, and used clustering to analyze “Book reading action patterns.” Goda et al. (2015) collected data from an e-learning system and used statistical methods to analyze the “Learning pace patterns”. This study used an e-book system to collect data, and the clustering method was applied to analyze “Backtrack reading patterns.” Junco and Clem (2015) found that students in the top 10th percentile in the number of highlights had significantly higher course grades than those in the lower 90th percentile. They also found that students who spent more time reading textbooks earned higher grades in the course than students who spent less time. Huang et al. (2016) proposed a Knowledge Tracing model that measures students’ level of knowledge on the underlying concept by looking at the amount of time s/he has spent on the related pages (e.g., read/skimmed). In addition, the correlation between students’ online reading behaviors in an e-book system and their academic achievement are investigated to identify the key e-book features that may affect students’ performance (Chen & Su, 2019) and engagement (Yang et al., 2020).

The operational behaviors of learning are diversified and vary with different operating objects. More specifically, E-learning behaviors include observable and hidden learning behaviors (Yin et al., 2019; Zhou et al., 2013). Observable learning behaviors can be easily and explicitly recorded and used for statistical analyses, such as pausing or making a marker. However, hidden learning behaviors cannot be observed and recorded in straightforward manners and needs to be inferred by using observable learning behaviors. For instance, “complete-jump behavior” in this study usually consists of multiple jump actions by a specific student, trying to find the right page to review. Therefore, it is difficult to observe directly and to determine how frequently they occur. However, learning outcomes are often affected by the visible measures and the implicit ones (Yin et al., 2019). We have to develop a model to extract those hidden learning behaviors from the dataset to investigate them.

While existing works provide access to learning log data, most of them aim to analyze or visualize observable learning behaviors rather than modeling implicit learning behaviors and studying them from different perspectives. However, our analysis differs because it focuses on modeling hidden user behaviors (jump-backs) in the educational context. It uses e-books to collect student learning log data from a real class setting.

## Method

### Dataset

The data used in this study were reading logs collected in an information science course at our university from October 2020 to February 2021. The course was offered to mostly first-year undergraduate students. A total of 225 students (mostly freshmen) attended the course. The learning goal of the course was to teach students the basic principles of information and communication technology, including information, calculation, communication, intelligence, and the algorithms of computer science.

Due to the Covid-19 situation in 2020, this lecture was conducted fully online via online meeting software (Zoom). The instructors and each student used their computers and an e-book system, BookRoll (Ogata et al., 2017), to access teaching slides that the instructor uploaded. The students were given the learning materials for the coming classes. They were free to access anytime and anywhere from a web browser on personal devices (computer, smartphone, etc.) for preparation or review purposes. Other learning activities, such as assignments, quizzes, forum discussions, and so on, were mainly conducted on a Moodle course page. The course lasts for 15 weeks, each class is 90-min long, and in the last week of the class, students took part in the final quiz examination.

**Fig. 2:**
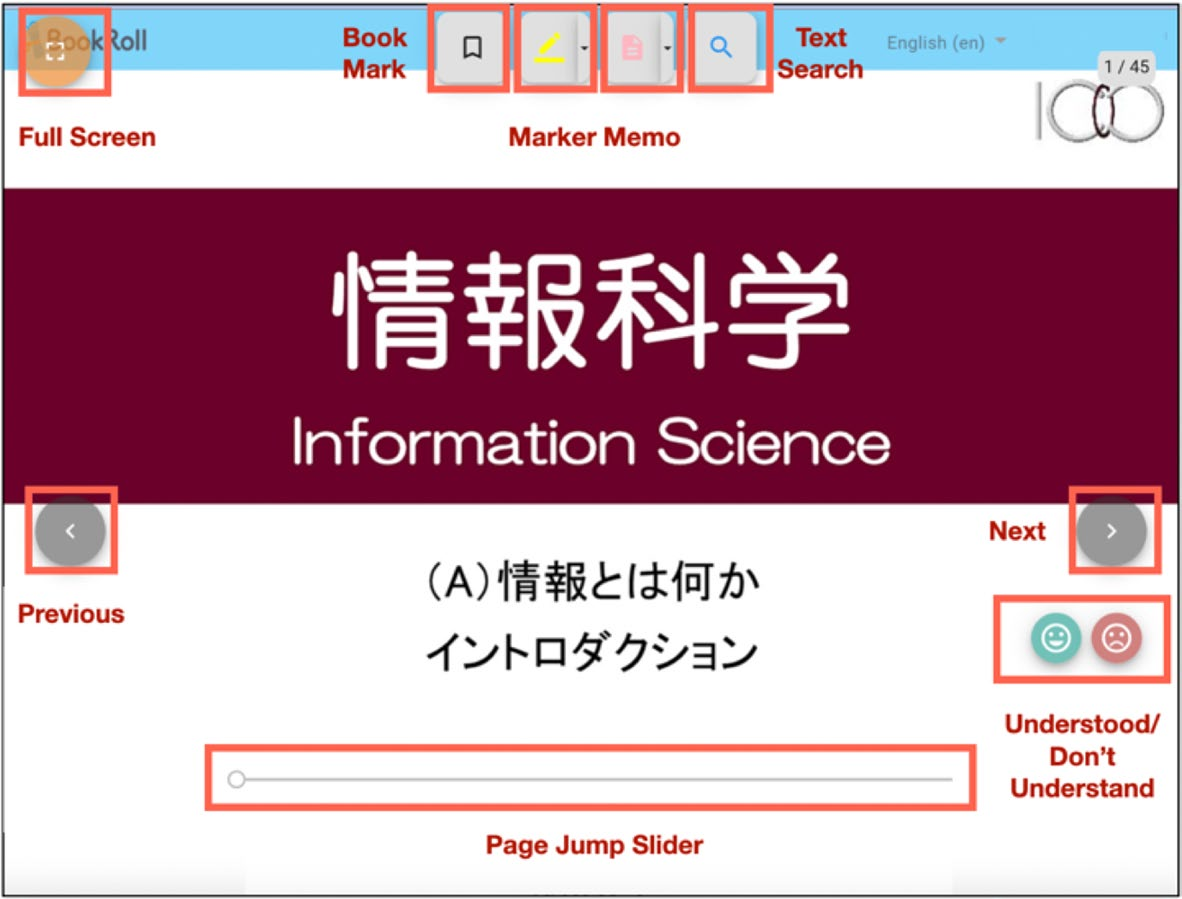
The screenshot of BookRoll interface

In the e-book system, BookRoll, there are features like bookmark, marker, memo annotating, etc. that students can use for learning (Fig. 2). All click-stream were recorded in a database that is related to students’ interaction with BookRoll. The collected click-stream data contained the following fields. *user id*: anonymized student user id. *operation name*: the action that was done, e.g., open, close, next, previous, jump, add markers, add bookmarks, etc. *page no*: the current page where the action was performed, *marker*: the reason for the marker added to a page, e.g., important, difficult, *role*: the role of the user, including student and teacher, *memo length*: the length of the memo that was written on the page, *device code*: the type of device used to view BookRoll, e.g., mobile, pc, and *operation date*: the timestamp of when the operation occurred.

There are different operations related to our research on page flipping, i.e., *PREV, NEXT, SEARCH JUMP, BOOKMARK JUMP*, and *PAGE JUMP*. *PREV* means that the student clicked the *previous* button to move to the previous page, and *NEXT* means that the student clicked the *next* button to move to the subsequent page. Students can also use a slider to change the page (*PAGE JUMP*), jump to a page from the list of the search result (*SEARCH JUMP*), or jump to a bookmarked page (*BOOKMARK JUMP*).

Table 1 lists the statistics of the event stream dataset recorded on a specific lecture slide. We found that *PAGE JUMP, SEARCH JUMP*, and *BOOKMARK JUMP* operations are rare in the dataset. Instead, students usually click the next or previous button quickly to jump to the desired page. For example, a student is on page 10 now, and he/she wants to jump to page 5, then he/she would like to click the *previous* button five times quickly instead of using the page slider (*PAGE JUMP*) function. The next section introduces our method to deal with such jump-back behaviors that are not explicitly reflected from the operation name field of the log data.

**Table 1:** Description of the event stream dataset for one of the lectures

Category	Type	Number
Student	Total Student #	225
Lecture	Lecture Time	90 min
	Page Length	41
Operation event	Total Event #	73,721
	Total PAGE JUMP #	1547
	Total SEARCH JUMP #	2
	Total BOOKMARK JUMP #	0
	Total NEXT #	48,113
	Total PREV #	22,341

### Jump back behavior modeling

To find out the jump-back behaviors from the timestamp-based click stream, we first define the concept complete-jump and other events of which a complete-jump consists.

#### **Definition 1**

*Complete-jump* A complete-jump consists of one (or multiple) jump actions by a specific student on a specific lecture slide, trying to find the right page to review. Let *(s,l,ps,pe)* denote a complete-jump, which means student *s* jumps back from start page *ps* to end page *pe* in slide *l* $(pe< ps)$.

#### **Definition 2**

*Jump back* (*Jb*) When a student uses the function that creates *PAGE JUMP, SEARCH JUMP*, or *BOOKMARK JUMP* log event from the current page (*ps*) to jump back to another page (*pe*) (*pe<ps*) or click the previous button to go to the previous page (*PREV* log event), then we say there is a jump back event. We noticed that a complete-jump might consist of more than one jump action. For example, the student clicks the *previous* button several times to jump back to a previous page. Another example, the student may jump back to a page of no interest and continue to look for the right page that she/he desires to review.

#### **Definition 3**

*Jump forward* (*Jf*) When a student uses the function that creates *PAGE JUMP, SEARCH JUMP*, or *BOOKMARK JUMP* log event from the current page (*ps*) to jump to a page afterward (*pe*) (*pe>ps*) or click the *next* button to go to the next page (*NEXT* log event), then we say there is a jump forward event. There also might be jump forward actions in a complete-jump. For example, the student jumps back too far away, and then she/he jumps forward to adjust to the correct position.

#### **Definition 4**

*Short-read* (*Sr*) After jumping to the desired page in the slide, the student would usually read for seconds. We name it a short-read event. We use the short-read event to determine the end of a complete-jump. Short-read is a duration period between two jump events, i.e., from the time the first jumping event ends (*t1*) to the time the next jumping event occurs (*t2*). i.e., *Sr=t2-t1*. According to the previous study (Yin et al., 2019), we tentatively set Sr to follow the rules below in our experiments.

*a.Invalid reading time* If a student spends less than 2 s on one page, then the student did not read the page.

*b.Invalid record* If the time difference between two neighboring log events is longer than 20 min, then the record is invalid. It means that the student is considered not reading the content of the page, as he/she did not conduct any action over 20 min.

Therefore, the duration period should be shorter than 20 min and longer than 2 s. i.e., *2* s ≤ *Sr* ≤ *20* min.

As we discussed, complete-jump behavior cannot be obtained straightforwardly. Enlighted by Zhang et al. (2017), we modified their algorithm based on deterministic finite automaton to reconstruct the behaviors. Based on the definitions above, we use a deterministic finite automaton (DFA) to construct the complete-jump behaviors.

**Fig. 3:**
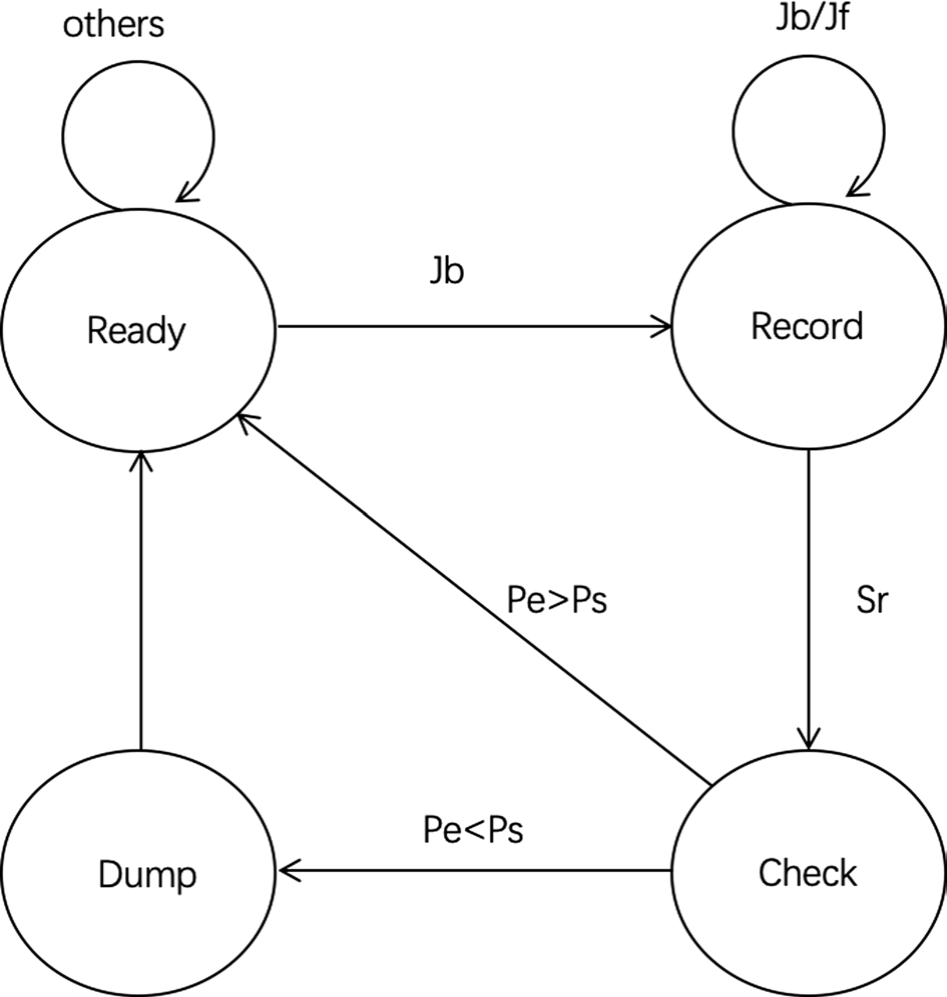
The construction of complete-jump behavior based on DFA

Figure 3 shows the state transition in the DFA. There are four states: Ready, Record, Check, Dump. At the Ready state, it stays until it receives a jump back event (*Jb*), then the state goes to Record. When the state is Record, it maintains a stack. When there are jump back events (*Jb*) or jump forward (*Jf*) events, it pushes all the events into the stack. If there comes a short-read event (*Sr*) or some other operations (e.g., the student uses *MAKER* or *MEMO* function), the state transforms to Check state. When the state is Check, it compares the start page (*ps*) of the event at the bottom of the stack and the end page (*pe*) of the event at the top of the stack. If *pe>ps*, the sequence of events in the stack constitutes a jump forward behavior, then the state goes back to Ready. Otherwise, the state transforms to Dump, where we aggregate the sequence of events in the stack to construct a complete-jump behavior.

**Fig. 4:**
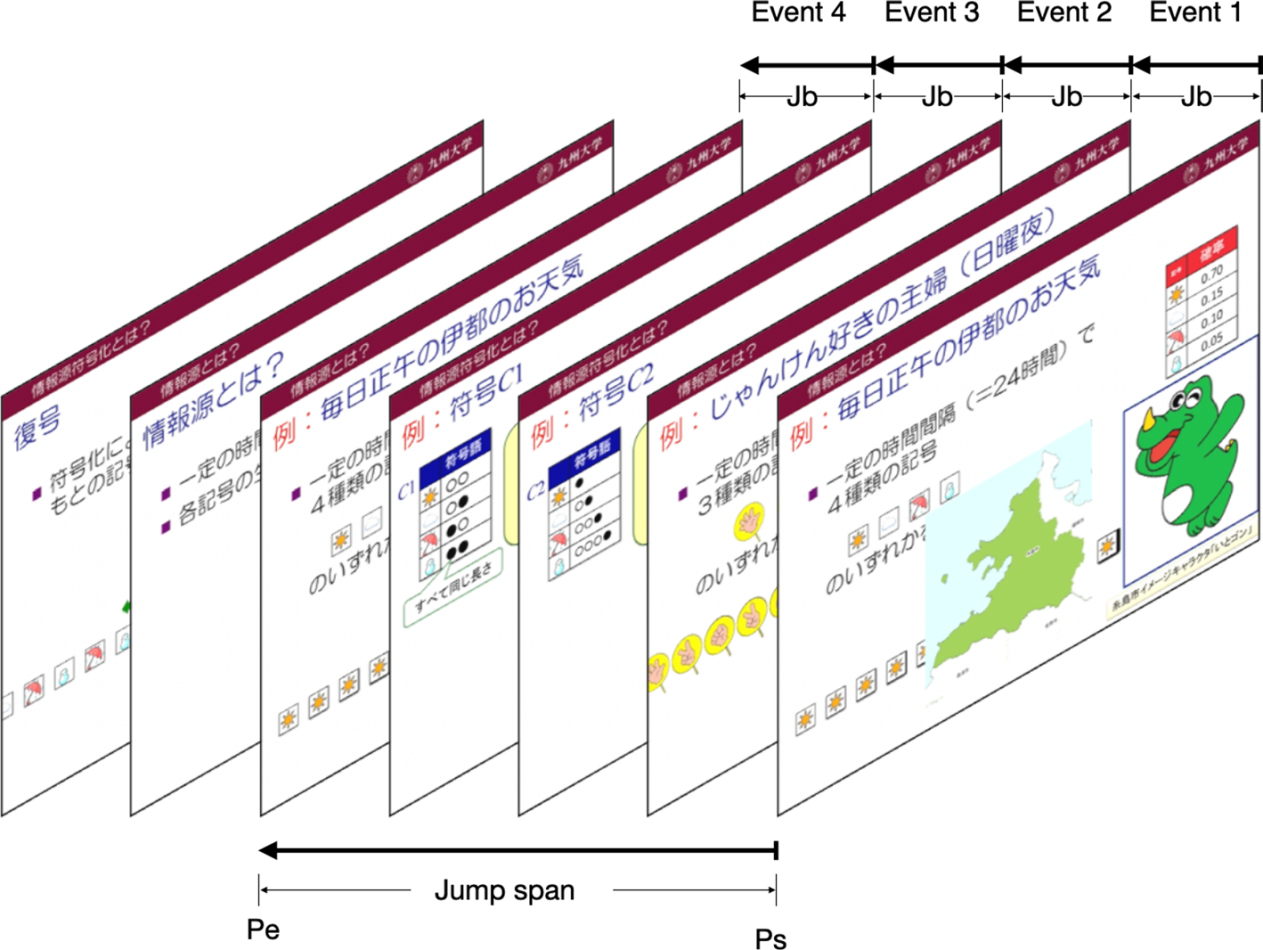
Complete-jump pattern A

**Fig. 5:**
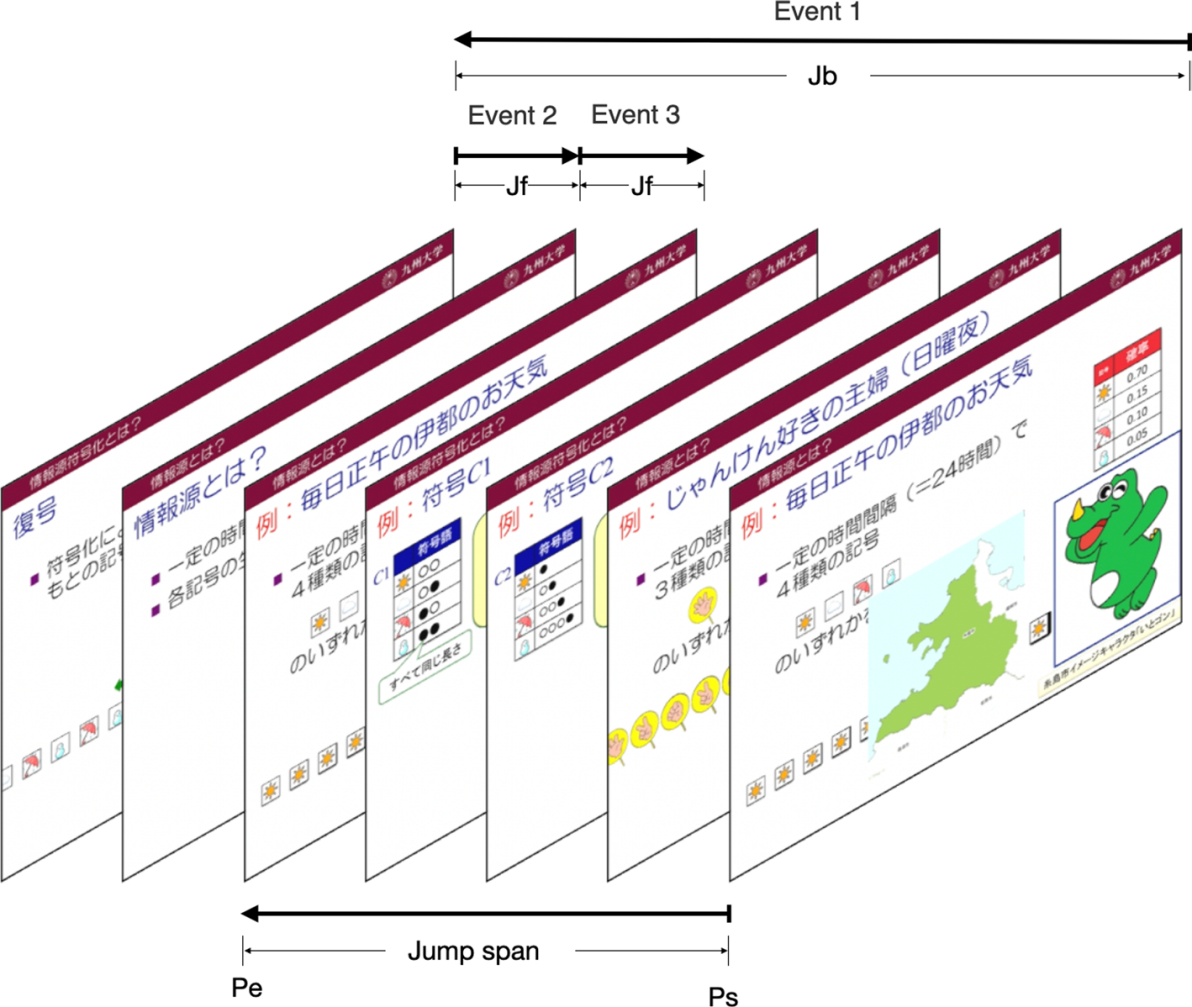
Complete-jump pattern B

Figures 4 and 5 show two common complete-jump patterns in the dataset. The pattern in Fig. 4 illustrates a kind of complete-jumps that consist of the event sequence (*Jb,Jb,Jb,Jb*), which means that the student uses the *previous* button four times to jump back to a previous page (*pe*). The pattern in Fig. 5 shows a complete-jump that consists of the event sequence (*Jb,Jf,Jf*). In this kind of scenario, the student uses *PAGE JUMP* operation to jump back to a previous page firstly and then clicks the *next* button two times to jump to a later page (*pe*).

Also, we name the number of pages between the start page and end page as a jump span. We can see that, although the patterns are different, these two complete-jumps have the same start page, end page, and jump span, and should be considered as the same complete-jumps.

### Data analysis

For the data analysis, we engaged in an investigation of the complete-jump behavior of the students. First, to better understand students’ in-class and out-class behaviors, we group students’ logs based on the operation time: (1) Preparation: the log data that student interacts with the learning materials before the class. (2) In class: the log data that student interacts with the learning materials during the class. (3) Review: the log data that student interacts with the learning materials after the class. Then, we analyzed our data by employing statistical analysis methods as well as the k-means clustering algorithm to answer our research questions. The details and results will be described in the following section.

## Result

### General characteristics

**Fig. 6:**
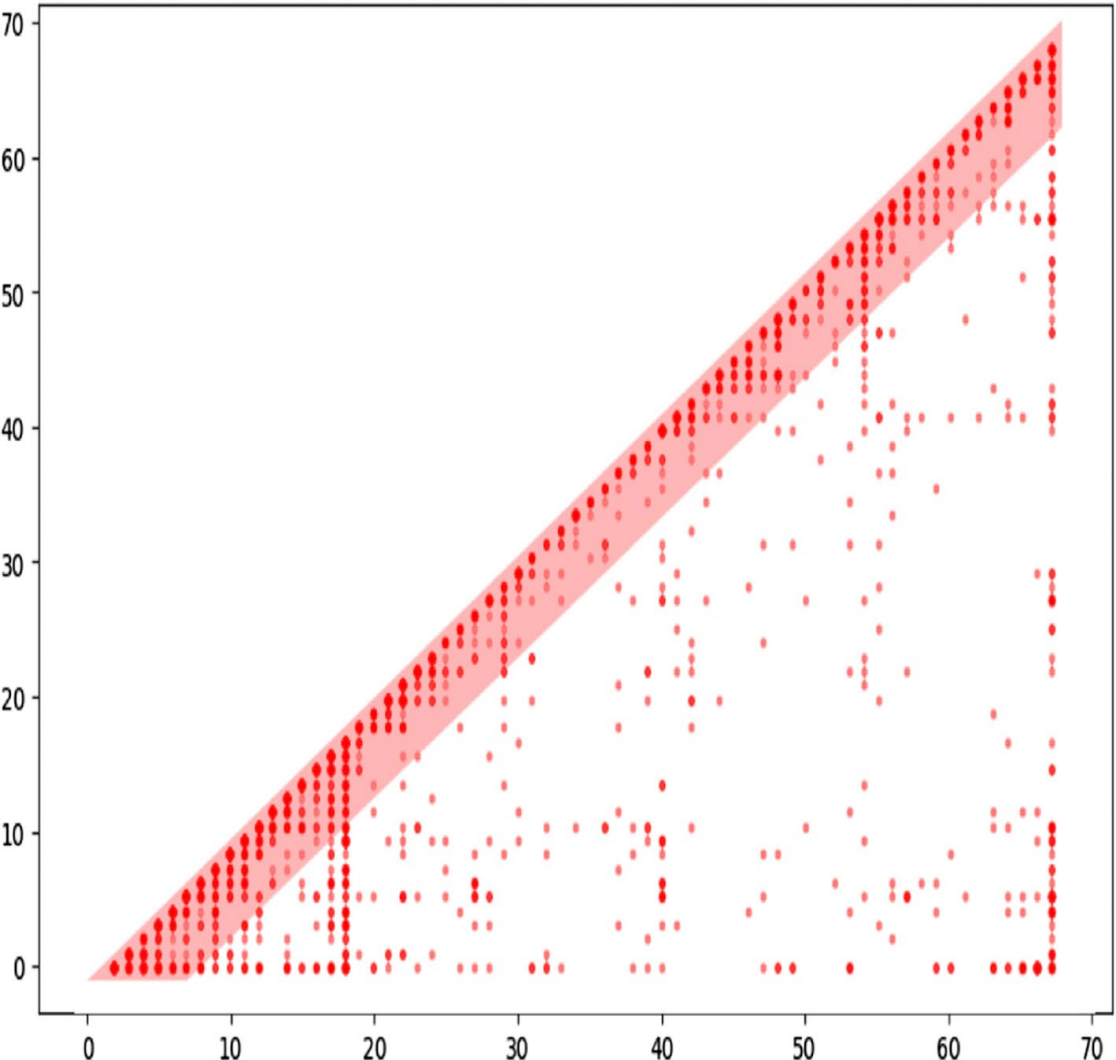
The scatter of complete-jumps. A spot at *(x,y)* represents a complete-jump from start page *x* to end page *y*

To better understand the general characteristics of students’ jump-back behaviors when reading e-books, we plot a scatter figure that shows all the complete jumps of the slide of a specific lecture in Fig. 6. The horizontal axis represents the start page of a complete jump back, and the vertical axis represents the end page of a complete jump back. Therefore, a spot (*x,y*) represents a complete-jump from the start page *x* to the end page *y*. The figure shows that most spots are near the diagonal. It indicates that students usually do not jump back to a more distant page from the current page. In this case, the jump span of 80% complete-jumps is smaller than six pages, shown as the red area. This phenomenon also exists in other lectures of the dataset.

**Fig. 7:**
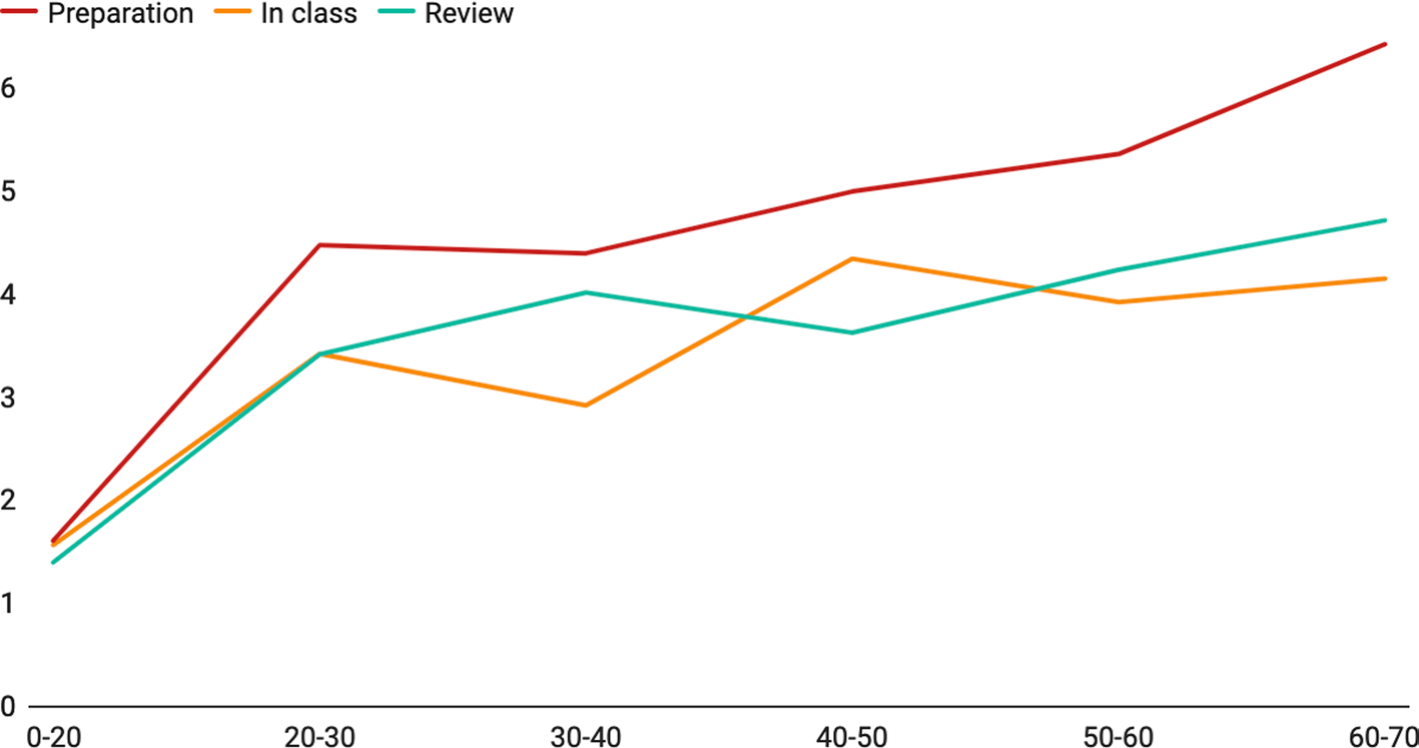
Correlation between jump spans and slide lengths in different lectures. Y-axis: average jump span of each group, X-axis: page length for each group

We have 22 learning materials in the dataset, and the page length for different learning materials varies a lot. We are interested in knowing whether the page length affects the complete-jump behavior. We group the slides by page length and examine the distribution of jump span and the total number of complete jumps in different slide groups. The result shown in Fig. 7 indicates that the jump span and the complete-jump number are positively correlated with the page length. We analyzed the difference between jump spans in and out of class using the paired sample t-test. The average jump span in preview activity is relatively larger than it during class (M_preview = 4.68, M_inclass = 3.35, *p* < 0.01) and after class (M_preview = 4.68, M_review = 3.82, *p* < 0.01). However, there is no significant difference between jump spans during and after class. It may be because students must follow the instructor during the class, and they are more familiar with the content of the course after the class. Therefore, the jump spans are relatively smaller compared to the preview period.

**Fig. 8:**
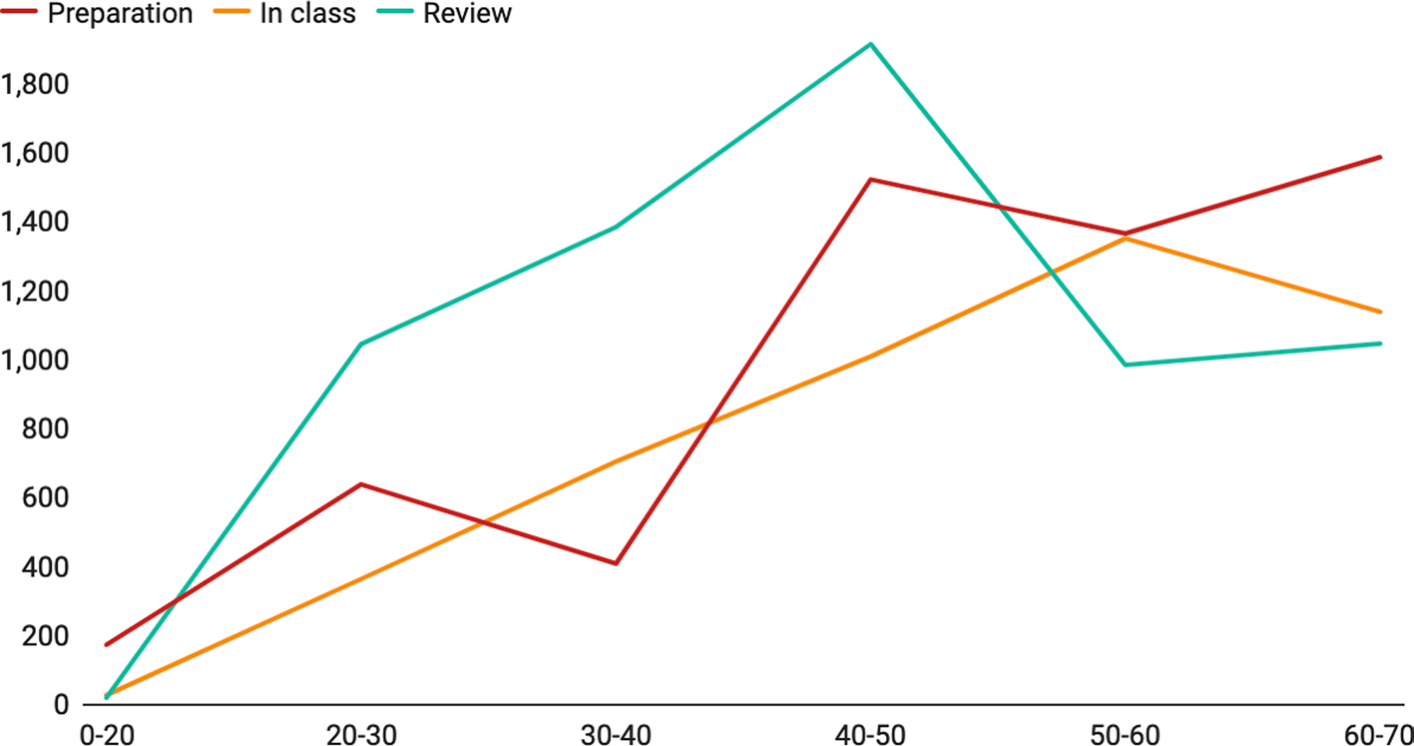
Correlation between the numbers of complete-jumps and page length in different lectures. Y-axis: average number of complete-jumps of each group, X-axis: page length for each group

Figure 8 shows the correlation between page length and the number of complete-jump. The result shows that the complete-jump number increases with the increase of page length at first then decreases. In general, the total complete-jump number in review activity (M_review = 1263.82) is relatively higher than it during class (M_inclass = 797.68) and preview (M_preview = 820.04). However, we find no significant difference in the total jump numbers in-class and out-class when analyzed using the paired sample t-test.

### Student preference of jump-back

Different students would have different jump-back patterns. For example, impatient students are likely to jump with higher frequency than patient students. To catch students’ preferences, we categorize students into different types based on their jump-back behaviors leveraging k-means clustering. The number of clusters is set to 3 based on the elbow method (Bholowalia & Kumar, 2014).

Table 2 shows the clustering results. It can be seen that students of clustering 1 (n = 21) have a clear preference when they jump back. They prefer to jump more times in and out of class with a shorter jump span, and they spend less time after jumping to their desired page. While students of cluster 2 (n = 159) prefer to jump back farther away with lower frequency, and they prefer to stay longer after jumping back to their desired page to have a serious reading. Another interesting phenomenon is about students in cluster 3 (n = 45). They seem to have no obvious preference during the class and the preparation. However, the frequency of their jump backs increases obviously in the review time, and the jump span also increases compared with the preparation and in-class.

**Table 2:** Clustering results of students’ jump back records

Type	Item	Description	C1	C2	C3
Preparation	# of Jump Back	Total number of jump back behaviors (in preparation)	**325.7**	48.3	119.3
	Average Jump Span	Average pages of jump span (in preparation)	3.7	**5.9**	4.1
	Average Stay Time(s)	Average time of reading after the student jumped back to the page (in preparation)	33.6	**62.7**	42.5
In class	# of Jump Back	Total number of jump back behaviors (in class)	**121.2**	66.3	99.6
	Average Jump Span	Average pages of jump span (in class)	3.1	**3.4**	3.2
	Average Stay Time(s)	Average time of reading after the student jumped back to the page (in class)	48.7	**54.2**	47.3
Review	# of Jump Back	Total number of jump back behaviors (in review)	209.4	71.2	**277.5**
	Average Jump Span	Average pages of jump span (in review)	3.7	**3.8**	**3.8**
	Average Stay Time(s)	Average time of reading after the student jumped back to the page (in review)	38.9	**40**	37.7

The largest value in each row is highlighted with bold text

### Jump-back and quiz score

As mentioned before, students took the quiz of the last lecture. We use the Pearson correlation coefficient (PCC) to calculate the partial correlation of quiz scores with other variables, such as the number of the jump back and jump span, etc. Table 3 presents the results of the partial correlation analysis. Pearson correlation coefficient results show that there are only weak correlations between several jump-back behaviors and the quiz score.

**Table 3:** Partial correlation results

Type	Item	Quiz Score	
		PCC	*p*-value
Preparation	# of Jump Back	0.27	*p* < 0.01
	Average Jump Span	0.07	0.30
	Average Stay Time(s)	0.17	*p* < 0.01
In class	# of Jump Back	0.35	*p* < 0.01
	Average Jump Span	0.19	0.03
	Average Stay Time(s)	0.10	0.13
Review	# of Jump Back	0.31	*p* < 0.01
	Average Jump Span	0.32	*p* < 0.01
	Average Stay Time(s)	0.21	0.02

**Fig. 9:**
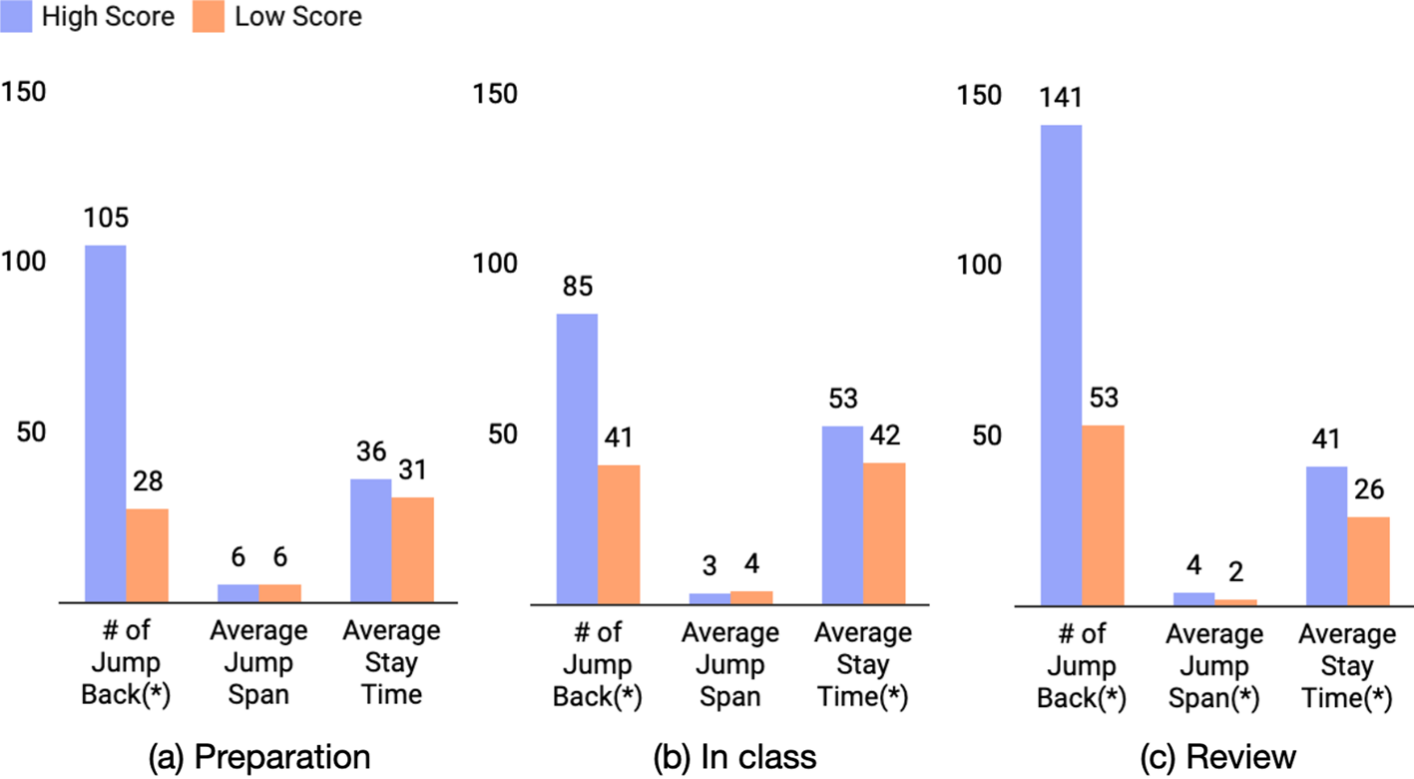
Comparison results between the high-score group and low-score group. (Significant Level (*) *p* < 0.01)

To provide a clear comparison, we split students into the high-score group (score over 90, N = 119) and low-score group (score below 80, N = 38) to compare the difference of jump-back related features in Fig. 9.

Based on the results, a safe conclusion could be drawn that compared with the high-score group, students with lower scores tend to have a significantly lower frequency of jump back and shorter stay time both in-class and out-class.

### Purposes for jump back

**Fig. 10:**
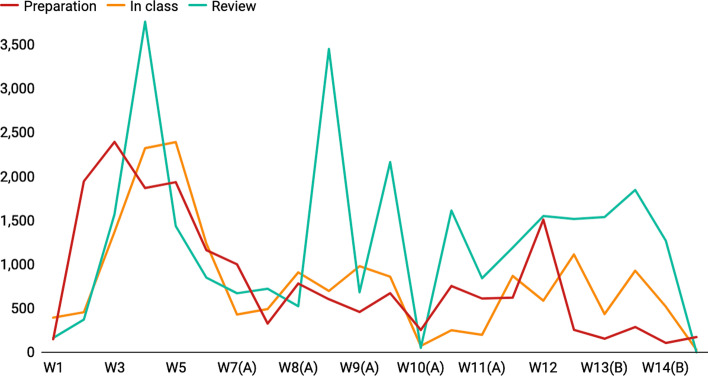
Number of complete-jumps across 14 weeks. Y-axis: number of complete-jumps, X-axis: weeks

**Fig. 11:**
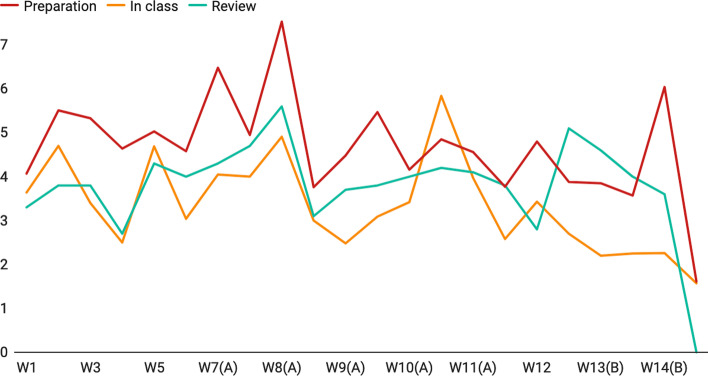
Average jump span across 14 weeks. Y-axis: average jump span, X-axis: weeks

The analysis explains the general performance of jump-back behaviors and how frequently they occur in different lectures, but it does not reveal why they occur. Figures 10 and 11 show the jump-back behaviors of all lectures across 14 weeks (Week 15 is removed because it is the final examination). The results indicate that jump-back behaviors might depend on many pedagogical methods such as the content of courses, problem sets, discussion, and exams.

**Fig. 12:**
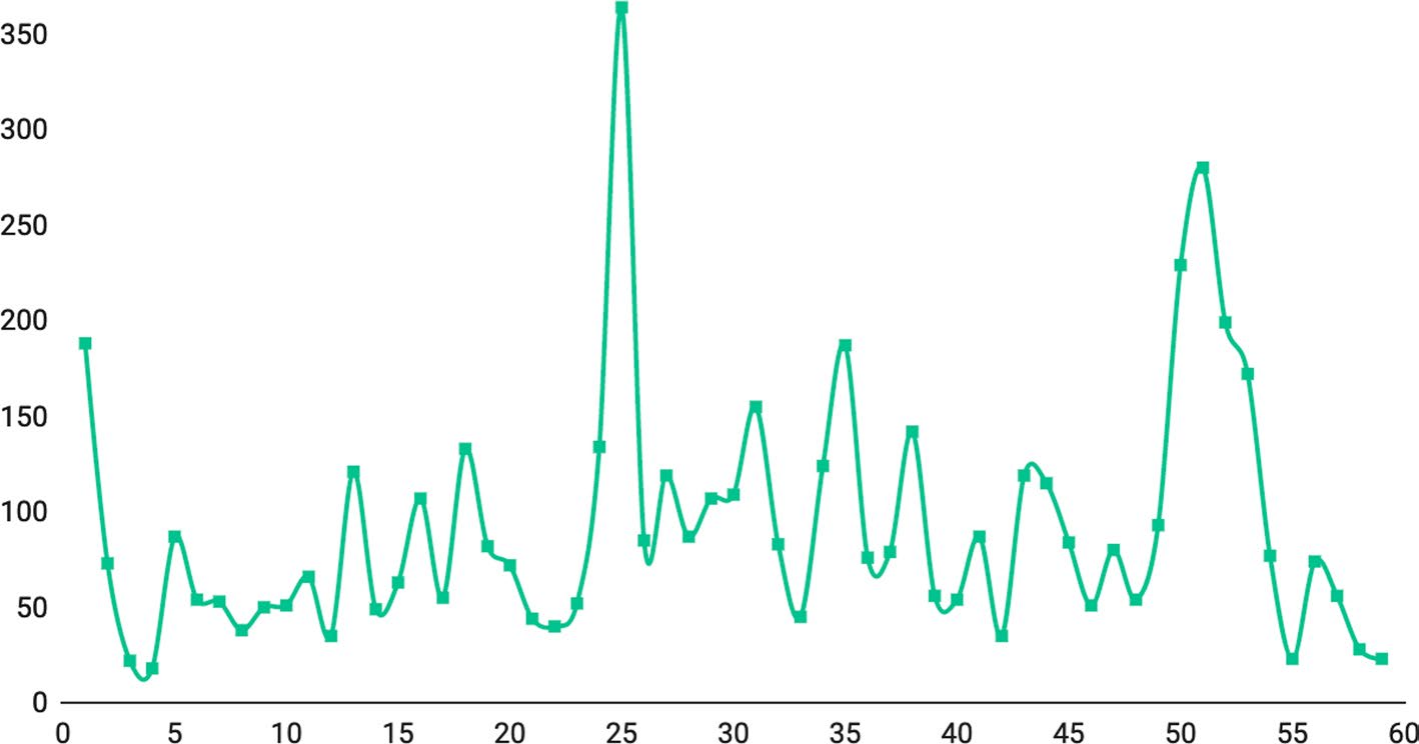
Number of complete-jumps in a lecture. Y-axis: total number of complete-jump to the page, X-axis: the page number of the lecture

To better understand the general jump back performance when reading e-books, we plot the number of complete jumps to each page of a lecture, as shown in Fig. 12. Y-axis is the total number of complete-jump to the page, and the x-axis is the page number. We can observe several pages with high jump back frequency in this lecture. Some e-books exhibited as many as 20 pages with high jump back frequency.

This subsection introduces a categorization of student jump-back behaviors by checking the actual content they read. While our categorization is not conclusive, it explains which semantic and contextual aspects of the slides might be responsible for a jump-back.

In the categorization process, we first read the e-books, especially paying attention to the detected pages with high jump back frequency. The goal was to group those pages into rough categories using the open card sorting method (Rugg & McGeorge, 1997). We discovered six groups in this generative process and named each. Then, we labeled all pages with high jump back frequency in the 22 learning materials to one of the categories generated in the first phase.

#### Categorization results

Overall, we discovered six groups as shown in Table 4. We will describe each category in the following subsections.

**Table 4:** Six slide types that students frequently jump back to

Slide page type	Percentage
Type 1. Explanation of concept and theorem	47
Type 2. Example problem and solution	20
Type 3. Assignments and In-class exercises	11
Type 4. Learning objectives	9
Type 5. Beginning of a new unit	9
Type 6. Tutorial steps	4

**Fig. 13:**
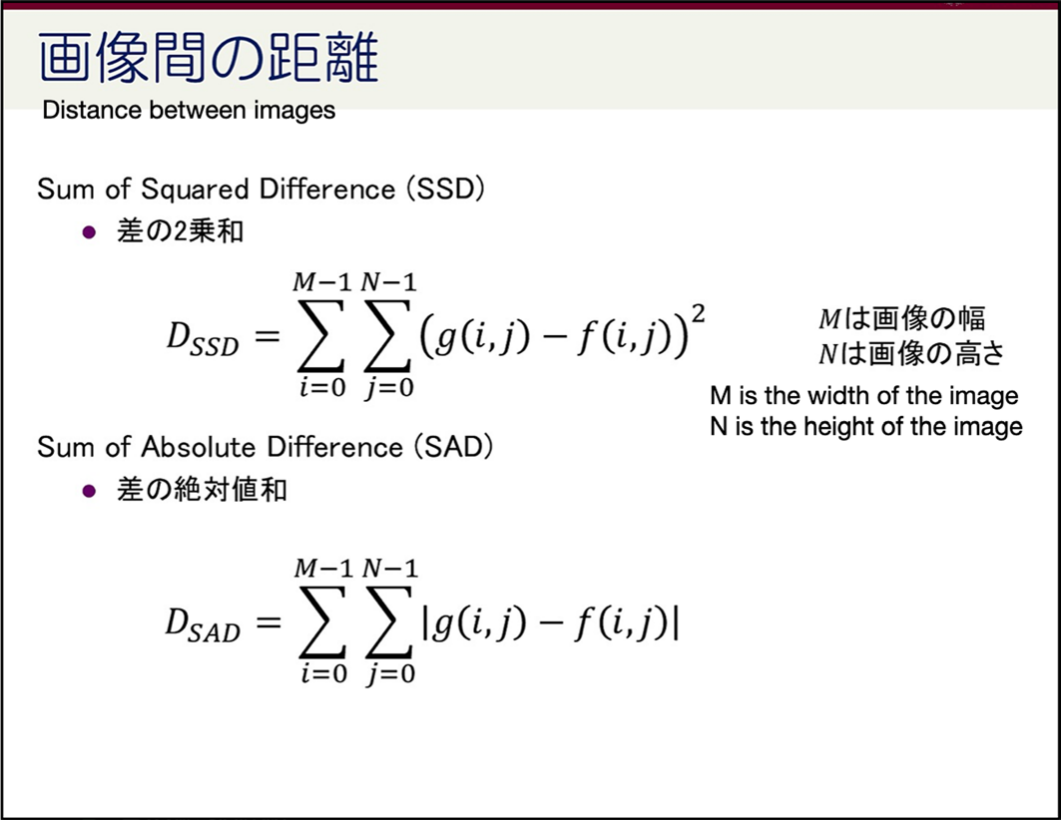
An example of Type 1 page: Explanation of concept and theorem

*Type1. Explanation of concept and theorem* Our results show that most pages belong to this category. It indicates that students are interested in the content of those pages, and they might want to review a confusing concept or revisit a mathematic equation when they need to use it. It might be important information for both instructors and students as those pages could be highlighted and summarized. Figure 13 shows an example from our course. A formal description of the concepts, their mathematic equations, and explanations is shown on this page.

**Fig. 14:**
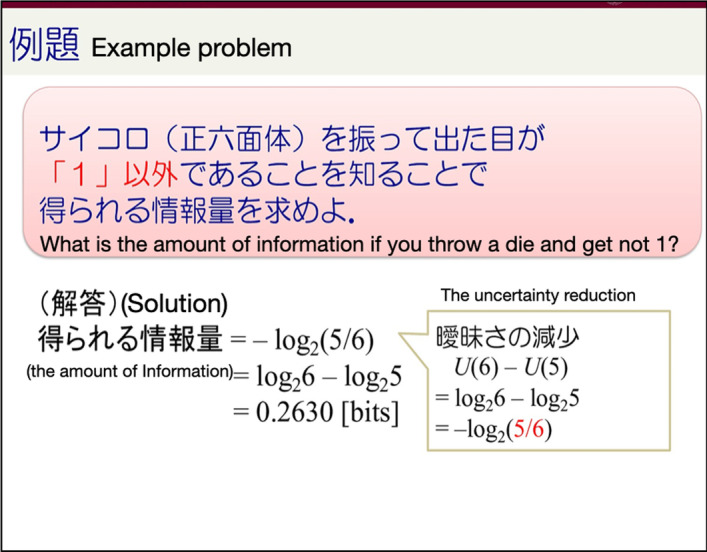
An example of Type 2 page: Example problem and solution

*Type2. Example problem and solution* In this category, students return to pages that show an example problem and the corresponding solution. By studying step-by-step solutions to solved problems, learning from examples might be the most effective way for students to learn new concepts. Figure 14 shows an example. Here the instructor explained the solution of a problem for calculating the amount of information after introduced information entropy theory, which was the main topic of the lecture. Moreover, this page got a lot of jump-back and attention, especially after class. Students may review those pages or referred to the example solution when they do their homework.

*Type3. Assignments and in-class exercises* In this category, students return to content that includes assignments and in-class exercises. This category is similar to type 2, but there is no solution or answer on such a page.

*Type4. Learning objectives* In this category, students jump back to a learning objective page. A potential interpretation is that students want to check the main topics and the progress of the lecture during the class. In the review process, they also check the learning objective page to ensure they do not miss important concepts. Although not every lecture of our class includes a learning objective page, students tend to have a high frequency to jump back to this page.

*Type 5. Beginning of a new unit* In this category, students browse to the beginning of a new unit, such as a new concept, example, or theorem. This category indicates that students are interested in reviewing a confusing concept from the beginning part or re-visit a theorem-proof sequence.

**Fig. 15:**
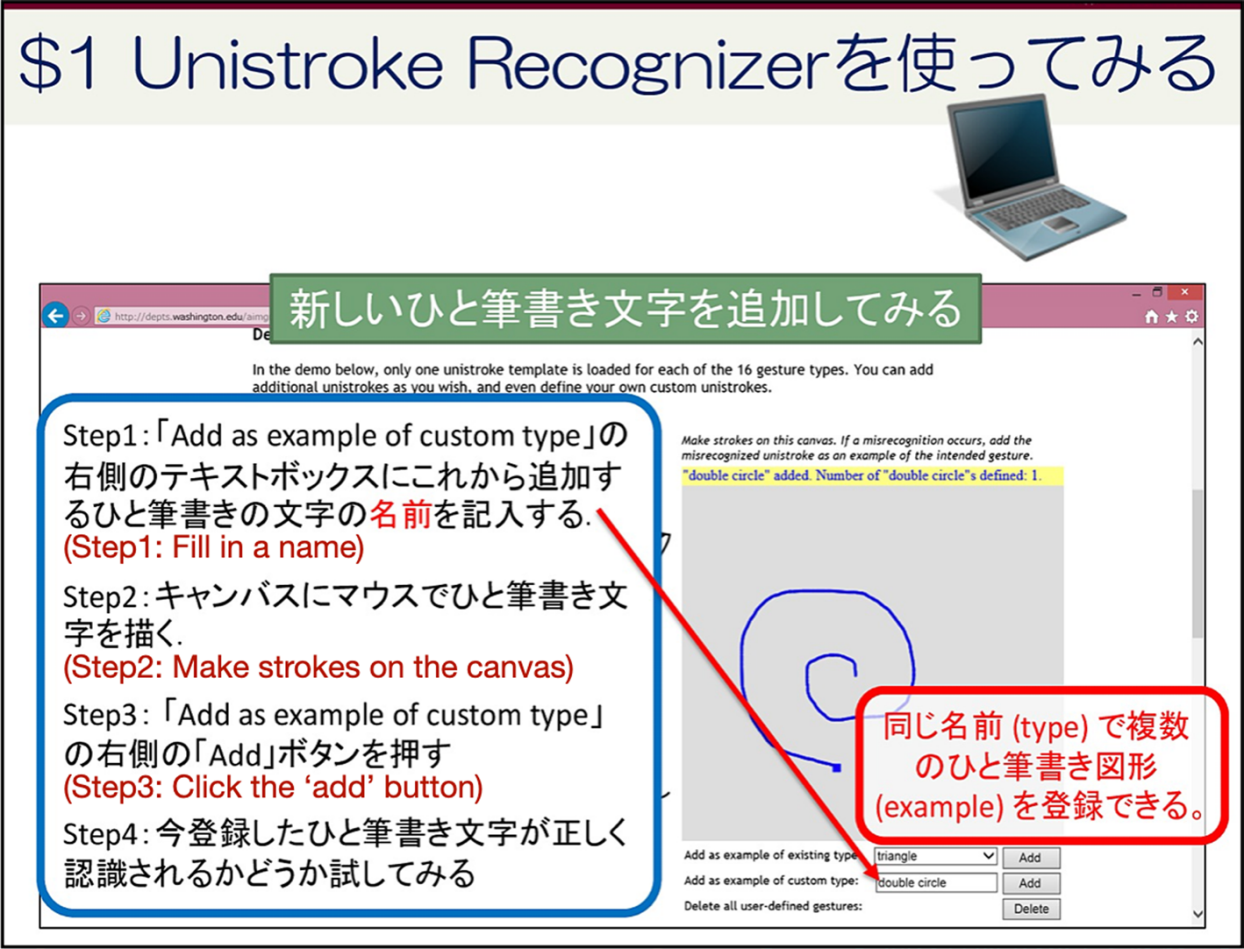
An example of Type 6 page: Tutorial steps

*Type 6. Tutorial steps* This category is students following the steps in the tutorial. While much less common than the other types, this type gives more specific information about student behavior. Tutorials often contain step-by-step instructions students can follow. We found that many students jump back and read the tutorial pages several times, possibly trying to replicate the step in their own tool. Figure 15 shows an example where the instructor introduces how to use a text recognition tool.

#### Comparison results

We compared those different types to see if there is any difference in terms of the number of jump-backs. Our results using the Mann-Whitney test showed significant differences between Type 2 and other types (*p* < 0.01), between Type 1 and Type 4 (*p* < 0.01), and between Type 1 and Type 5 (*p* < 0.01). As shown in Table 5, this suggests that Type 2 were significantly got more attention from students than other types. Also, students jump to Type 1 with a higher frequency than jump to Type 4 and Type 5 significantly. There was no significant difference found for other types.

**Table 5:** Slide types statistics

Slide page type	# of Jump Back Mean (SD)
Type 1. Explanation of concept and theorem	154.9 (95.2)
Type 2. Example problem and solution	277.0 (177.7)
Type 3. Assignments and In-class exercises	136.9 (99.8)
Type 4. Learning objectives	100.6 (75.3)
Type 5. Beginning of a new unit	148.8 (70.7)
Type 6. Tutorial steps	89.8 (36.6)

## Discussion

### Answering the research questions

First, the results of our studies indicate that students usually do not jump back to a distant page (more than six pages) from the current one. Besides, the jump span and the complete jump number is positively correlated with the length of the slides. There is also some interesting phenomenon when we investigate the difference of jump-back behaviors in and out of class (RQ1).

Different students would have different jump-back patterns, and we employ the k-means clustering algorithm to identify three different types of students in terms of jump-back behaviors. Students of Type 1 prefer to jump more times in and out of class with a short jump span and stay short time after jumping to their desired page. Students of Type 2 would like to jump back farther away with lower frequency, but they prefer to stay longer after jumping back to have a serious reading. Students of Type 3 seem to have no obvious preference during the class and the preparation. However, the frequency of their jump backs becomes obviously higher in the review time, and the jump spans also become longer compared with the preparation and in-class (RQ2).

Compared with the high-score group, students with low scores tend to have a significantly lower frequency of jump back and shorter stay time both in-class and out-class (RQ3). This finding shows that student academic achievement is strongly affected by reading behaviors, particularly by returning to relevant pages, and is in accordance with previous works (Chen et al., 2021).

Finally, we discovered six slide categories that are responsible for jump backs. That is, explanation of concept and theorem, example problem and solution, assignments and in-class exercise, learning objectives, beginning of a new unit, and tutorial steps (RQ4).

With the insight of the dataset focusing on the jump-back behaviors, we found there can be opportunities to benefit the improvement of the instruction designs and learning strategies with the in-depth analysis of jump-back behaviors. From a learner perspective, analyzing the timing and target pages of the jump-backs is possible to find out the students’ common problems in understanding the content during class time. By comparing the differences of jump-back behaviors between in and out of classes, as well as between high-score and low-score groups, it is possible to find out suggestions of relevant pages (e.g., to certain exercises) and even better learning styles (Boticki et al., 2019; Gyllen et al., 2018). From an instructor perspective, this study makes a step forward towards understanding the importance of jump-back behaviors and how they are related to the student’s performance and course content. The findings of this study can improve course designs, teaching materials, and the structure of the e-books. For example, based on “Backtrack Reading” information, instructors can confirm the teaching contents and make changes, such as adding more explanations. Based on page jump information, they can improve their teaching materials by changing the order of the pages or adding the link to these two pages (Yin et al., 2015b).

### Reading path dashboard

Our approach for modeling jump behaviors can be used in visual tools to provide both instructors and learners with richer data about click-level e-book interaction.

**Fig. 16:**
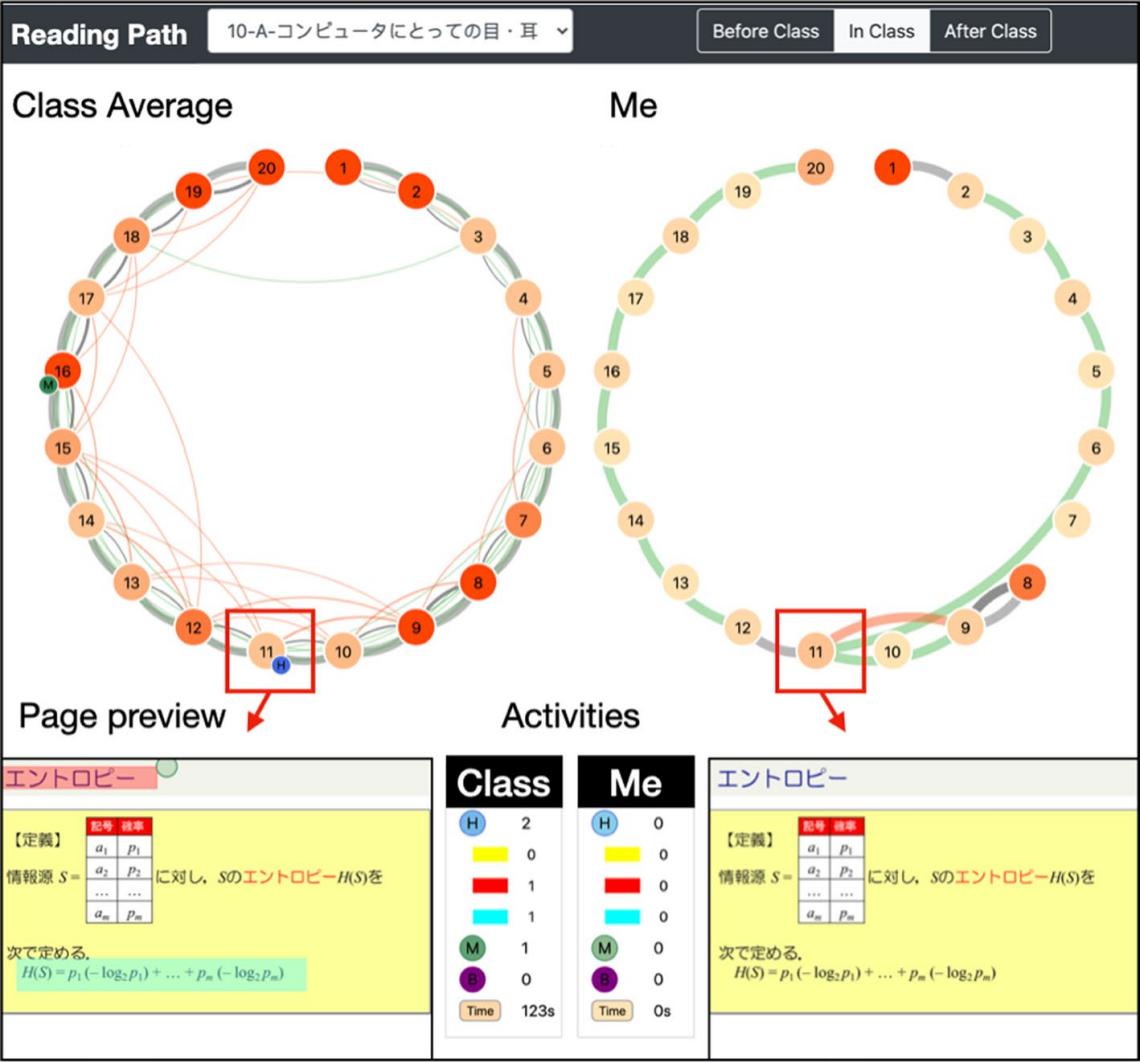
The reading path dashboard prototype

Reading path dashboard (Lu, et al., 2020) is an example to support the exploration of e-book interaction data together with our data-driven method, as shown in Fig. 16. The left is the visualization of jump behaviors as the reading path for all students of the class and the right is the visualization of the reading path for a selected student. The nodes on a circle represent pages and the links between the nodes represent the reading path. The intensity of a node’s color indicates the reading time spent on the page, and the thickness of a link shows the number of the page transit. The smaller circles attached to a page node present the recorded learning behaviors, including highlight markers and memo annotations on the page. When the learner clicks a page node in the overview, the page preview, as well as the details of the reading time and learning behaviors will be displayed. Such a dashboard that visualizes the jump behaviors can support instructors make data-driven decisions about planning and editing their learning materials. Also, they can use it to respond to students’ interests and confusion while a course is being offered. On the other hand, it helps learners be aware of their learning processes and regulates their learning strategies.

The dashboard enables visually connecting e-book content with salient patterns in the graph. We expect to support the sensemaking process for course instructors, e-learning system designers, and students.

### Limitations

While our analysis methods identified e-book interaction behaviors, understanding why we see these behaviors is difficult. Log stream data cannot accurately represent students’ real intent (e.g., jump to a page but not read). Furthermore, presentation-quality or storyline might also affect which parts of the learning material students jump back to read. While it is difficult because the class is fully conducted online, incorporate such data may extract a more comprehensive understanding of the jump-back behaviors. Finally, our analysis does not consider different learners in the class. Per-group analysis of our techniques might help us better reason about the results.

## Design implications

The analysis of students’ behaviors introduced in this paper can guide the design of better e-book learning experiences. Our analysis shows that students interact with e-books differently, depending on the pedagogical and stylistic properties of the content. We argue that course instructors, e-learning system designers, and students can benefit from such information. We present a set of design implications from our analysis results.

*Utilize jump information* Our results suggest that pages with important content get many jump backs. The information can be used to mark highlights in the slide automatically. Visualize those data in e-learning systems could help instructors respond to students’ interests and confusion. In addition, it can be used to effectively summarize highlights from a slide, which can be useful for students to access these important pages without having to rely on imprecise seeking.

*Provide interactive links and navigations* Providing interactive links and navigations might reduce the navigation overhead for students. Our observation suggests that many students jump back to an early page to review a concept or a solution example related to the current exercise page. Interactive links to these pages can be useful for students willing to read them repeatedly, which is time-saving and convenient compared with the current *BOOKMARK JUMP* function and *SEARCH JUMP* function. Another potential option is providing multiple screens or even multiple devices, showing the concept page in one and the exercise page in another. Many video interfaces provide navigation distribution according to the historical data (Carlier et al., 2011; Kim et al., 2014a; Yadav et al., 2015), and then detect user intention and suggest potential positions to go automatically. We consider that interactive links and navigations are also necessary for e-book systems.

*Consider making learning objectives and summarization page* An interesting phenomenon in our results is that many students tend to jump back to a learning objective page, especially in their review time. For course instructors and slide designers, showing the content structure of the lecture and making the summarization page of each lecture is an important point.

## Conclusion

This research aims to tackle specific types of reading behaviors of learners while using the e-book system to seek a better understanding of how students read and learn. Particularly, this paper provides an in-depth look into how students interact with digital textbooks and studies the students’ intentions for jump-back behaviors.

Through the analytics of e-book event stream data, we at first formally define the “jump-back” behaviors and extract them from the click event stream of slide reading, then systematically studied the jump-back behaviors from different perspectives. Finally, we provide explanations of which semantic and contextual aspects of the slide are responsible for jump backs. We suggest our data-driven analytic methods can help improve the e-book learning experience and informs design implications for future learning materials and e-book systems.

For the next steps, we plan to analyze more courses, data streams, and interaction patterns. We hope to analyze the differences in the general jump-back patterns between different courses. For example, compare the results between humanities courses and science courses. Another potential work is to take the teaching processes into account, which can be helpful to construct better explanatory models of irregular page-viewing behaviors. Furthermore, it will invoke new feedback approaches to the instructors and students, for example, suggestions of useful jump-back destinations of the current page, which will contribute to the improvement of learning efficiency.

## Data Availability

Data and material are not available as our consent forms did not include information regarding sharing data outside of the research study.

## Abbreviations

DFA: Deterministic Finite Automaton
Jb: Jump back
Jf: Jump forward
LMSs: Learning Management Systems
PCC: Pearson Correlation Coefficient
Sr: Short read

## References

[CR2] Akçapinar, G., Chen, M.-R. A., Majumdar, R., Flanagan, B., & Ogata, H. (2020). Exploring student approaches to learning through sequence analysis of reading logs. In *Proceedings of the tenth international conference on learning analytics & knowledge* (pp. 106–111).

[CR1] Akçapınar, G., Hasnine, M. N., Majumdar, R., Flanagan, B., & Ogata, H. (2019). Developing an early-warning system for spotting at-risk students by using ebook interaction logs. *Smart Learning Environments,**6*(1), 4.

[CR3] Avlonitis, M., & Chorianopoulos, K. (2014). Video pulses: User-based modeling of interesting video segments. *Advances in Multimedia,**2014,* 2.

[CR4] Bholowalia, P., & Kumar, A. (2014). EBK-means: A clustering technique based on elbow method and k-means in WSN. *International Journal of Computer Applications,**105*(9), 17–24.

[CR5] Boticki, I., Akçapınar, G., & Ogata, H. (2019). E-book user modelling through learning analytics: The case of learner engagement and reading styles. *Interactive Learning Environments,**27*(5–6), 754–765.

[CR6] Brinton, C. G., & Chiang, M. (2015). MOOC performance prediction via clickstream data and social learning networks. In *2015 IEEE conference on computer communications (INFOCOM)* (pp. 2299–2307). IEEE.

[CR7] Carlier, A., Ravindra, G., Charvillat, V., & Ooi, W. T. (2011). Combining content-based analysis and crowdsourcing to improve user interaction with zoomable video. In *Proceedings of the 19th ACM international conference on multimedia* (pp. 43–52).

[CR8] Chen, C.-H., & Su, C.-Y. (2019). Using the BookRoll e-book system to promote self-regulated learning, self-efficacy and academic achievement for university students. *Journal of Educational Technology& Society,**22*(4), 33–46.

[CR9] Chen, C.-H., Yang, S. J., Weng, J.-X., Ogata, H., & Su, C.-Y. (2021). Predicting at-risk university students based on their e-book reading behaviours by using machine learning classifiers. *Australasian Journal of Educational Technology,**37,* 130–144.

[CR10] Cheng, K.-H., & Tsai, C.-C. (2014). Children and parents’ reading of an augmented reality picture book: Analyses of behavioral patterns and cognitive attainment. *Computers& Education,**72*, 302–312.

[CR11] Chorianopoulos, K. (2013). Collective intelligence within web video. *Human-centric Computing and Information Sciences,**3*(1), 1–16.

[CR12] Chorianopoulos, K., Leftheriotis, I., & Gkonela, C. (2011). SocialSkip: Pragmatic understanding within web video. In *Proceedings of the 9th European conference on interactive TV and video* (pp. 25–28).

[CR13] Costa, A. L., & Kallick, B. (2008). *Learning and leading with habits of mind: 16 essential characteristics for success*. ASCD.

[CR14] Crossley, S., Paquette, L., Dascalu, M., McNamara, D. S., & Baker, R. S. (2016). Combining click-stream data with NLP tools to better understand MOOC completion. In *Proceedings of the sixth international conference on learning analytics & knowledge* (pp. 6–14).

[CR15] Freeman, R. S., & Saunders, E. S. (2016). E-book reading practices in different subject areas: An exploratory log analysis. In S. M. Ward, R. S. Freeman, & J. M. Nixon (Eds.), *Academic E-Books* (p. 223). Purdue University Press.

[CR16] Goda, Y., Yamada, M., Kato, H., Matsuda, T., Saito, Y., & Miyagawa, H. (2015). Procrastination and other learning behavioral types in e-learning and their relationship with learning outcomes. *Learning and Individual Differences,**37*, 72–80.

[CR17] Gyllen, J., Stahovich, T., & Mayer, R. (2018). How students read an e-textbook in an engineering course. *Journal of Computer Assisted Learning,**34*(6), 701–712.

[CR18] Huang, Y., Yudelson, M., Han, S., He, D., & Brusilovsky, P. (2016). A framework for dynamic knowledge modeling in textbook-based learning. In *Proceedings of the 2016 conference on user modeling adaptation and personalization* (pp. 141–150).

[CR19] Junco, R., & Clem, C. (2015). Predicting course outcomes with digital textbook usage data. *The Internet and Higher Education,**27*, 54–63.

[CR20] Kim, J., Guo, P. J., Cai, C. J., Li, S.-W., Gajos, K. Z., & Miller, R. C. (2014a). Data-driven interaction techniques for improving navigation of educational videos. In *Proceedings of the 27th annual ACM symposium on user interface software and technology* (pp. 563–572).

[CR21] Kim, J., Guo, P. J., Seaton, D. T., Mitros, P., Gajos, K. Z., & Miller, R. C. (2014b). Understanding in-video dropouts and interaction peaks in online lecture videos. In *Proceedings of the first ACM conference on learning@ scale conference* (pp. 31–40).

[CR22] Law, E.L.-C., & Lárusdóttir, M. K. (2015). Whose experience do we care about? Analysis of the fitness of Scrum and Kanban to user experience. *International Journal of Human-Computer Interaction,**31*(9), 584–602.

[CR23] Li, N., Kidziński, Ł, Jermann, P., & Dillenbourg, P. (2015). MOOC video interaction patterns: What do they tell us? In G. Conole, T. Klobucar, C. Rensing, J. Konert, & E. Lavoué (Eds.), *Design for teaching and learning in a networked world* (pp. 197–210). Spain: Springer.

[CR24] Lindsey, R. V., Shroyer, J. D., Pashler, H., & Mozer, M. C. (2014). Improving students’ long-term knowledge retention through personalized review. *Psychological Science,**25*(3), 639–647. 24444515 10.1177/0956797613504302

[CR25] Liu, D.Y.-T., Bartimote-Aufflick, K., Pardo, A., & Bridgeman, A. J. (2017). Data-driven personalization of student learning support in higher education. In A. Peña-Ayala (Ed.), *Learning analytics: Fundaments, applications, and trends* (pp. 143–169). Springer.

[CR26] Lorenzen, S., Hjuler, N., & Alstrup, S. (2018). Tracking behavioral patterns among students in an online educational system. In *International educational data mining society*.

[CR150] Lu M, Chen L, Goda Y, Shimada A, Yamada M (2020) In *Development of a learning dashboard prototype supporting meta-cognition for students*. Companion Proceedings of the 10th International Conference on Learning Analytics \& Knowledge (LAK20), (pp. 104–106)

[CR27] Ma, B., Chen, J., Li, C., Liu, L., Lu, M., Taniguchi, Y., & Konomi, S. (2020). Understanding jump back behaviors in e-book system. In *Companion proceedings of the 10th international conference on learning analytics & knowledge* (pp. 623–631).

[CR28] McKay, D. (2011). A jump to the left (and then a step to the right) reading practices within academic ebooks. In *Proceedings of the 23rd Australian computer–human interaction conference* (pp. 202–210).

[CR29] Mostow, J. (2004). Some useful design tactics for mining its data. In *Proceedings of the ITS2004 workshop on analyzing student–tutor interaction logs to improve educational outcomes* (pp. 20–28).

[CR30] Myrberg, C. (2017). Why doesn’t everyone love reading e-books? *Insights the UKSG Journal,**30*(3), 115–126.

[CR31] Ogata, H., Oi, M., Mohri, K., Okubo, F., Shimada, A., Yamada, M., et al. (2017). Learning analytics for e-book-based educational big data in higher education. In H. Yasuura, C. M. Kyung, Y. Liu, & Y. L. Lin (Eds.), *Smart sensors at the IoT Frontier* (pp. 327–350). Springer.

[CR32] Oi, M., Okubo, F., Shimada, A., Yin, C., & Ogata, H. (2015). Analysis of preview and review patterns in undergraduates’ e-book logs. In *Proceedings of the 23rd international conference on computers in education* (pp. 166–171).

[CR33] Okubo, F., Yamashita, T., Shimada, A., & Ogata, H. (2017). A neural network approach for students’ performance prediction. In *Proceedings of the seventh international learning analytics & knowledge conference* (pp. 598–599).

[CR34] Rainie, L., Zickuhr, K., Purcell, K., Madden, M., & Brenner, J. (2012). *The rise of e-reading*. Pew Internet & American Life Project.

[CR35] Ren, Z., Uosaki, N., Kumamoto, E., Liu, G.-Z., & Yin, C. (2017). Improving teaching materials through digital book reading log. In *Proceedings of the international conference on advanced technologies enhancing education* (pp. 90–96).

[CR36] Rugg, G., & McGeorge, P. (1997). The sorting techniques: A tutorial paper on card sorts, picture sorts and item sorts. *Expert Systems,**14*(2), 80–93.

[CR37] Shepperd, J. A., Grace, J. L., & Koch, E. J. (2008). Evaluating the electronic textbook: Is it time to dispense with the paper text? *Teaching of Psychology,**35*(1), 2–5.

[CR38] Shimada, A., Okubo, F., & Ogata, H. (2016). Browsing-pattern mining from e-book logs with non-negative matrix factorization. In *EDM* (pp. 636–637).

[CR39] Shin, J. (2012). Analysis on the digital textbook’s different effectiveness by characteristics of learner. *International Journal of Education and Learning,**1*(2), 23–38.

[CR40] Sutcliffe, A., & Hart, J. (2017). Analyzing the role of interactivity in user experience. *International Journal of Human-Computer Interaction,**33*(3), 229–240.

[CR41] Taniguchi, Y., Shimada, A., Yamada, M., & Konomi, S. (2019). Recommending highlights on students’ e-textbooks. In *Society for information technology & teacher education international conference* (pp. 1128–1134). Association for the Advancement of Computing in Education (AACE).

[CR42] Wang, G., Zhang, X., Tang, S., Zheng, H., & Zhao, B. Y. (2016). Unsupervised clickstream clustering for user behavior analysis. In *Proceedings of the 2016 CHI conference on human factors in computing systems* (pp. 225–236).

[CR43] Yadav, K., Shrivastava, K., Mohana Prasad, S., Arsikere, H., Patil, S., Kumar, R., & Deshmukh, O. (2015). Content-driven multi-modal techniques for non-linear video navigation. In *Proceedings of the 20th international conference on intelligent user interfaces* (pp. 333–344).

[CR44] Yang, A., Chen, Y., Flanagan, B., & Ogata, H. (2020). Applying key concepts extraction for evaluating the quality of students’ highlights on e-book. In *28th international conference on computers in education conference proceedings* (Vol. 1, pp. 284–288). Asia-Pacific Society for Computers in Education (APSCE).

[CR45] Yin, C., Okubo, F., Shimada, A., Kojima, K., Yamada, M., Fujimura, N., & Ogata, H. (2014). Smart phone based data collecting system for analyzing learning behaviors. In *International conference on computer in education (ICCE 2014)* (pp. 575–577).

[CR47] Yin, C., Okubo, F., Shimada, A., Oi, M., Hirokawa, S., & Ogata, H. (2015a). Identifying and analyzing the learning behaviors of students using e-books. In *Proceedings of the 23rd international conference on computers in education* (pp. 118–120). Asia-Pacific Society for Computers in Education Hangzhou, China.

[CR46] Yin, C., Okubo, F., Shimada, A., Oi, M., Hirokawa, S., Yamada, M., Kojima, K., & Ogata, H. (2015b). Analyzing the features of learning behaviors of students using e-books. In *Proceedings of the international conference on computers in education* (pp. 617–626).

[CR48] Yin, C., Yamada, M., Oi, M., Shimada, A., Okubo, F., Kojima, K., & Ogata, H. (2019). Exploring the relationships between reading behavior patterns and learning outcomes based on log data from e-books: A human factor approach. *International Journal of Human-Computer Interaction,**35*(4–5), 313–322.

[CR49] Zhang, H., Sun, M., Wang, X., Song, Z., Tang, J., & Sun, J. (2017). Smart jump: Automated navigation suggestion for videos in MOOCS. In *Proceedings of the 26th international conference on world wide web companion* (pp. 331–339).

[CR50] Zhou, Z.-J., Hu, C.-H., Zhang, B.-C., Xu, D.-L., & Chen, Y.-W. (2013). Hidden behavior prediction of complex systems based on hybrid information. *IEEE Transactions on Cybernetics,**43*(2), 402–411. 22907969 10.1109/TSMCB.2012.2208266

